# Dataset of xenobiotics human renal clearance values

**DOI:** 10.1093/database/baag018

**Published:** 2026-04-11

**Authors:** Natalia Łapińska, Sebastian Polak

**Affiliations:** Chair of Pharmaceutical Technology and Biopharmaceutics, Faculty of Pharmacy, Jagiellonian University Medical College, Medyczna 9, 30-688 Kraków, Poland; Chair of Pharmaceutical Technology and Biopharmaceutics, Faculty of Pharmacy, Jagiellonian University Medical College, Medyczna 9, 30-688 Kraków, Poland; Certara UK Ltd (Simcyp Division), 1 Concourse Way, Sheffield S1 2BJ, United Kingdom

## Abstract

Scientific articles have been searched for experimentally established critical pharmacokinetic parameter—renal clearance. The main source of the documents was PubMed database, considered one of the most important and comprehensive repositories of biomedical literature. After manual data collection and thorough quality check database presenting human renal clearance values of exogenous substances was developed. After collecting the data, preliminary processing and simple analysis were carried out. The final database contains over 1700 experimental observations from 761 scientific articles and covers over 500 unique substances studied. **Database URL:** doi: https://10.17632/3427x3wzzc.2

## Introduction

The human body is a complex, dynamic biological system in which numerous physiological processes and organ systems interact to maintain homeostasis—internally stable conditions despite external and internal changes [1, 2]. Homeostasis is a dynamic and continuous process, and the regulation of many parameters requires active coordination of organ systems [2, 3]. The key organ involved in maintaining homeostasis is the kidneys, which play a central role in regulating body fluid composition, electrolyte balance, acid-base balance, and the removal of metabolic waste products and toxins [1, 4, 5].

Renal clearance (CLren) is a basic parameter of kidney function. CLren reflects the efficiency of renal excretion and represents the theoretical volume of plasma from which a substance is completely removed and excreted into the urine per unit of time [6]. CLren depends on three main processes: glomerular filtration, tubular secretion, and reabsorption, which determine the rate of substance flow between blood and urine. In a healthy body, glomerular filtration rate averages 120–150 ml/min and is the main factor determining the clearance of endogenous substances such as creatinine and inulin [7]. In principle, renal clearance is estimated based on the relation between the rate of drug excreted in urine and its concentration in plasma over time [8]. It requires urine collection over time and parallel determination of drug concentration in urine and plasma. Detailed description of methods used to calculate CLren for drugs, physiological and physicochemical determinants of renal clearance has been provided by Tucker [9].

In drug pharmacokinetics, renal clearance plays a significant role in influencing the drug’s exposure and thus, its therapeutic activity and potential safety threats. This parameter is crucial in determining the dose, especially in patients with renal impairment, in whom drug elimination may be slowed down [10]. In addition, the kidneys contain numerous transporters responsible for the active secretion and reabsorption of drugs [11]. In case of polytherapy, an interplay between substrates and perpetrators can lead to clinically significant interactions between drugs competing for the same transport system [12]. Understanding these mechanisms is essential when designing new molecules and evaluating their pharmacokinetics (PK), efficacy, and safety profile.

Noting the lack of a publicly available database containing information on drugs CLren, we decided to fill this gap. This article presents a method for collecting and cleaning the database, which resulted in the development of a final, reliable database of xenobiotics and their corresponding total renal clearance values. The presented database will enable the use of collected information for data analysis and potentially the development of predictive models. It should be noted that such models, namely empirical predictors calculating human renal clearance for xenobiotics, exist and can be used [13, 14]. They, however, share a common weakness—no access to original data used for model development. This prohibits verification of the modelling step and thus adds to the reproducibility crisis [15]. Our aim is to avoid such situations and promote open and thus reliable, reproducible and replicable science.

## Materials and method

The research material consisted of scientific articles presenting experimental data on the human renal clearance values of exogenous substances. The primary platform for searching for this data was PubMed [16]. Specific limiting criteria were adopted in the data collection process: only data from adult humans were included. Only substances introduced directly into the body were analyzed, omitting information on their metabolites. The database does not contain information on multiple drugs taken simultaneously. The main recorded data include the dose administered, the route of administration, and the analytical method used to assess the amount of substance excreted in the urine. In addition, the average body weight of the participants, the number of individuals involved in the study, and their health status were included. Only values representing total renal clearance of the parent compound after single-dose administration were included; data derived under steady-state conditions were excluded to ensure methodological consistency.

During the subsequent data-cleaning phase, the Simplified Molecular Input Line Entry System (SMILES) notation was introduced for each molecule using information from the PubChem database [17]. Molecules for which structural data were not available were excluded. Introducing SMILES notation also enabled the standardization of substance names—from those used in the original research to those currently accepted [e.g. Ro 17-2301 (AMA-1080) → Carumonam; S-9780 → Perindoprilat; PF-06650833 → Zimlovisertib]. During this name-cleaning step, nomenclature of well-known substances was also standardized; e.g. acetaminophen was adopted as the preferred name for paracetamol in the database. Another factor reducing the number of records was ambiguity regarding substance doses.

To standardize the dataset, we adopted litres per hour (l/h) as the primary unit of clearance. In some articles, renal clearance was reported in units normalized to body surface area (ml/min/1.73 m² or l/h/1.73 m²). These values were converted based on the average body weight and height of the study group or their reported body surface area. If these data were unavailable, standard values were used: 70 kg for body weight and 170 cm for height. This procedure was necessary for 42 clearance records originating from 15 publications. As individual anthropometric data were not reported in these studies, the use of standard values may introduce some uncertainty in the converted estimates due to potential differences between assumed and actual body size distributions in the respective populations.

## Results

PubMed database contains over eight thousand articles that include the keyword ‘renal clearance’. After applying filters to limit the results to studies involving adult humans, original research articles, and works published in English, approximately two thousand articles remained.

In the next phase, the criteria described in the *Materials and methods* section were applied. An additional important criterion was the availability of full-text articles. At this stage, 802 articles met the above-mentioned requirements.

**Figure 1:**
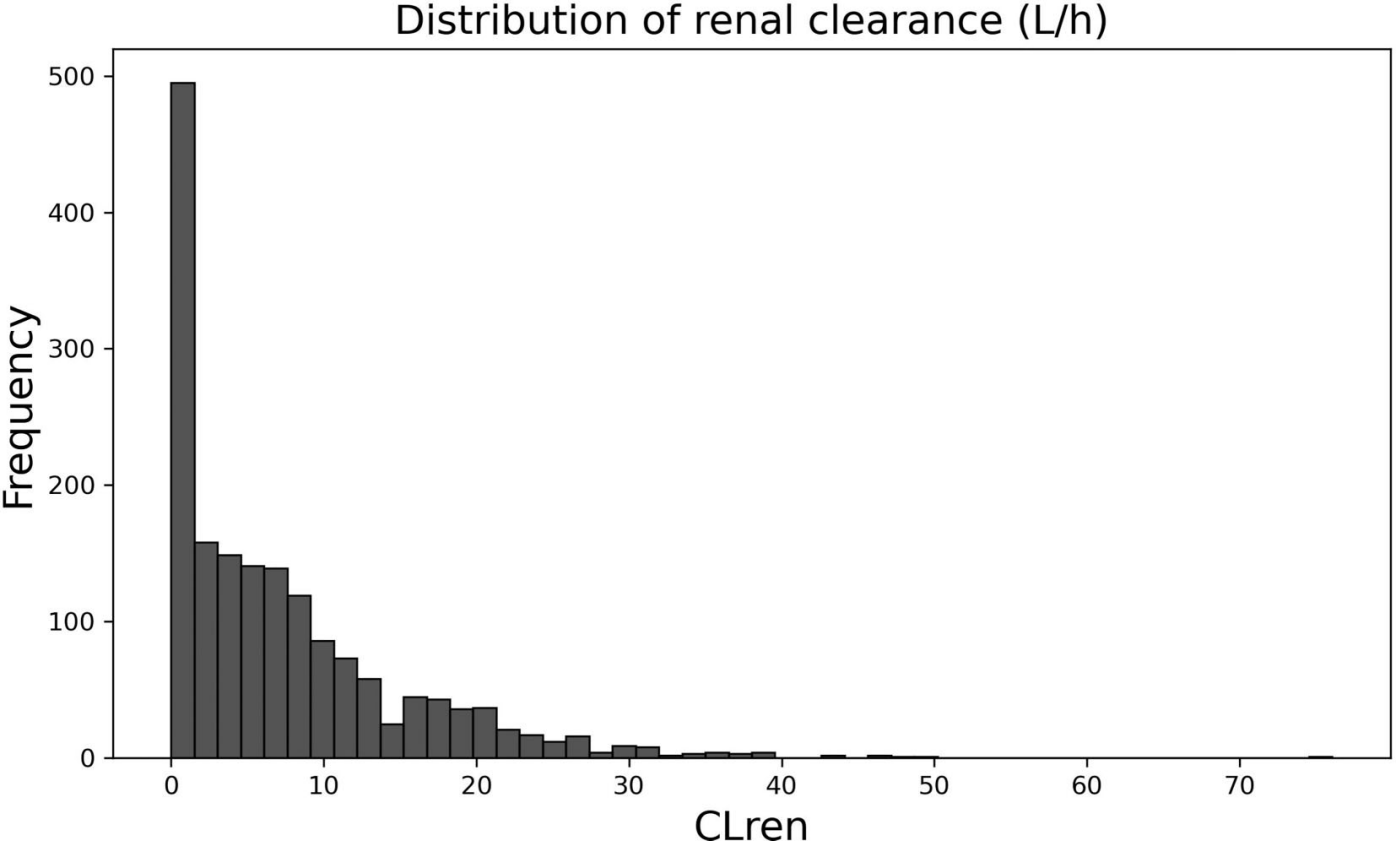
Histogram of renal clearance values (L/h).

Ultimately, the database included 761 scientific articles and consists of 1714 experimental CLren values for 508 unique chemicals. The distribution of renal clearance values is presented in Fig. 1.

**Figure 2:**
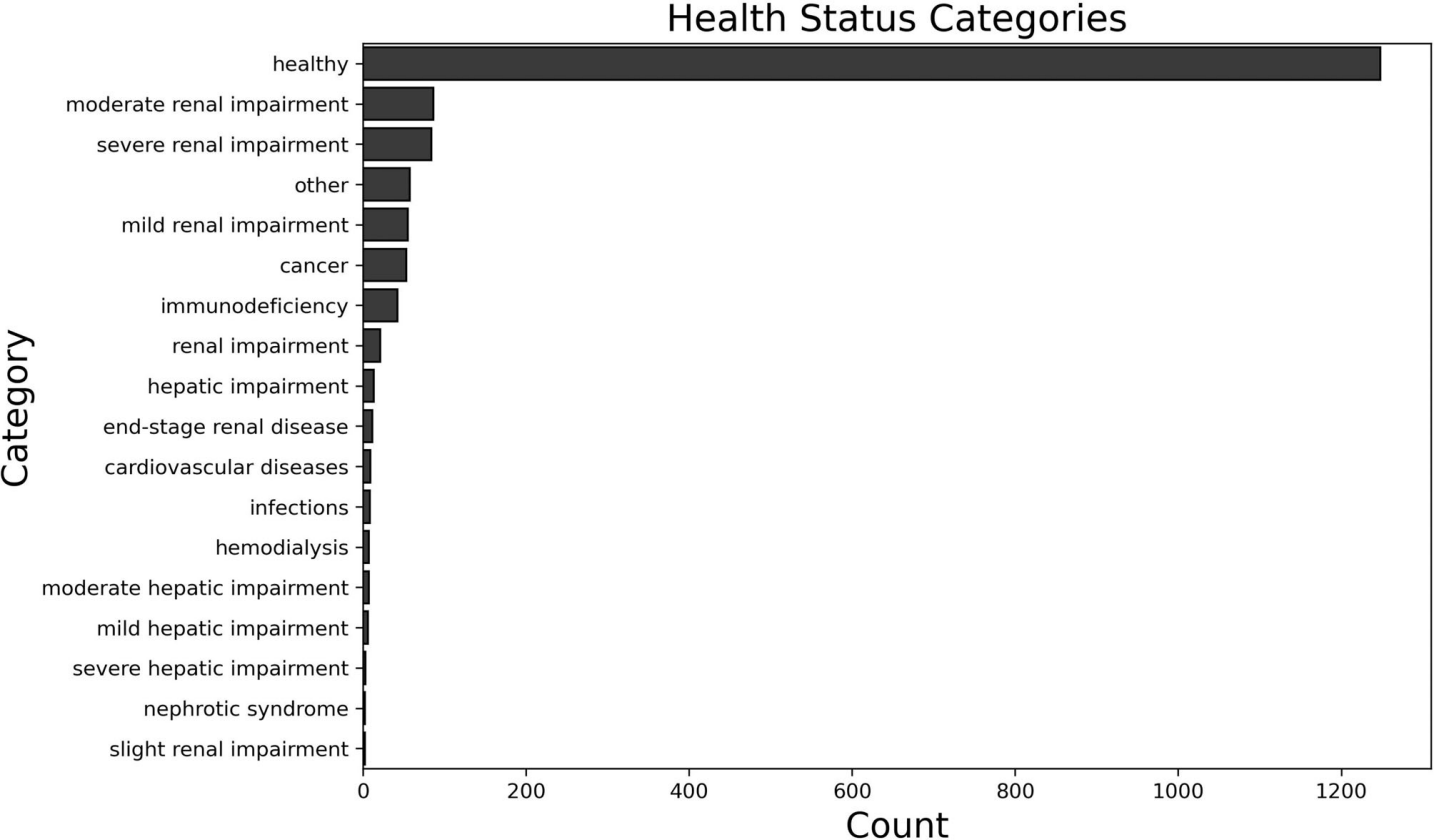
Categories of health status and their counts.

The structure of the database with respect to health status is presented below. Seventy-three percent of the data was obtained from healthy volunteers’ studies (1248). Another major group consisted of individuals with varying degrees of renal impairment, categorized as mild (55), moderate (86), severe (84), end-stage renal disease [16], and general descriptions of renal dysfunction [15]. The distribution of these categories is shown in Fig. 2. In total, there are 260 measurements of renal clearance in individuals with impaired kidney function. Other significant groups include participants with hepatic impairment (29 records), various forms of cancer (53), and those with autoimmune diseases, immune deficiencies, infections, and several single-entry conditions grouped as ‘other’.

**Figure 3:**
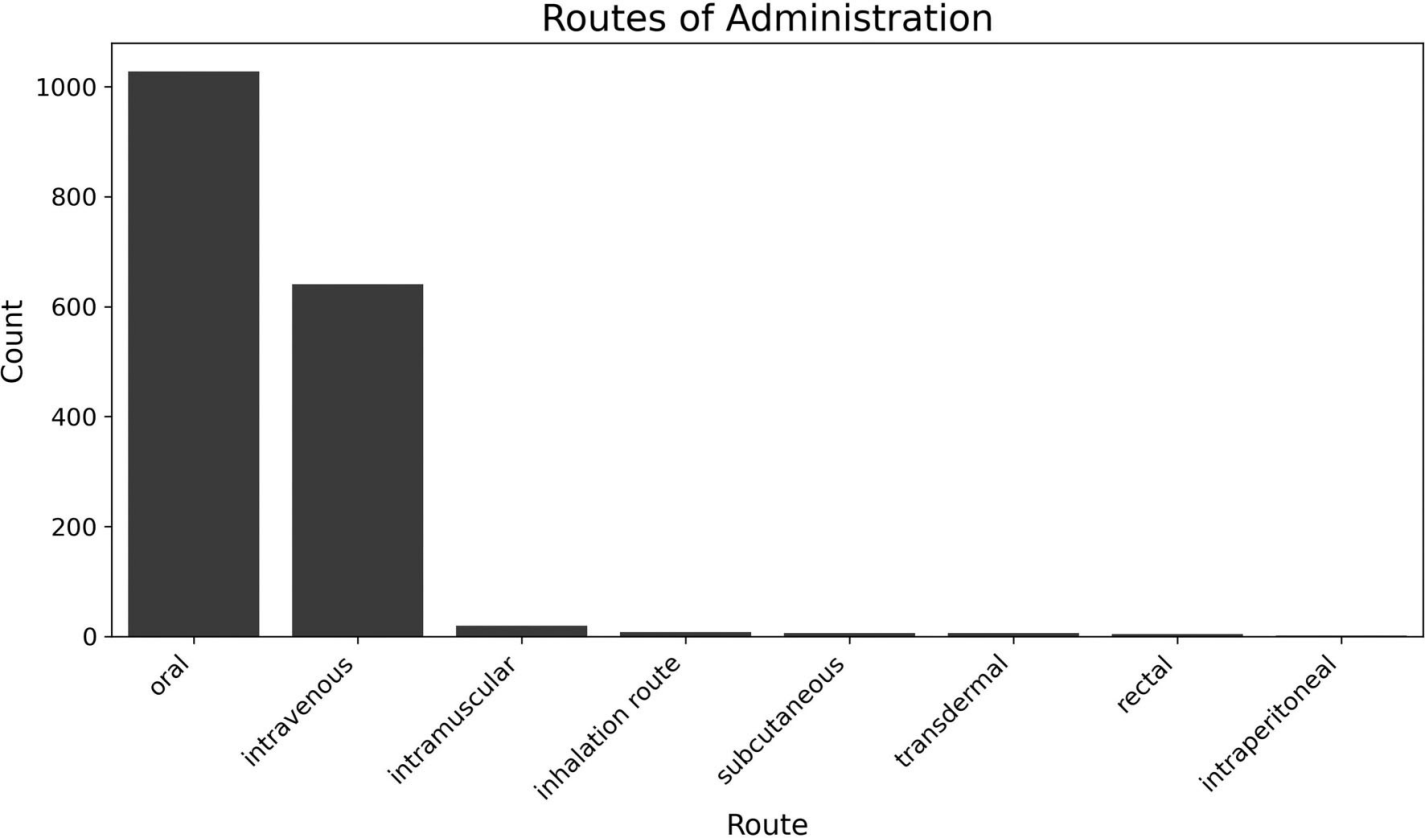
Histogram showing the frequency with which a particular route of administration was chosen for a drug.

An important aspect of the studies included in the analysis was the route of drug administration. The database contains seven routes of administration, with the most common being intravenous and oral administration. A bar chart of all routes of administration is shown in Fig. 3.

**Figure 4:**
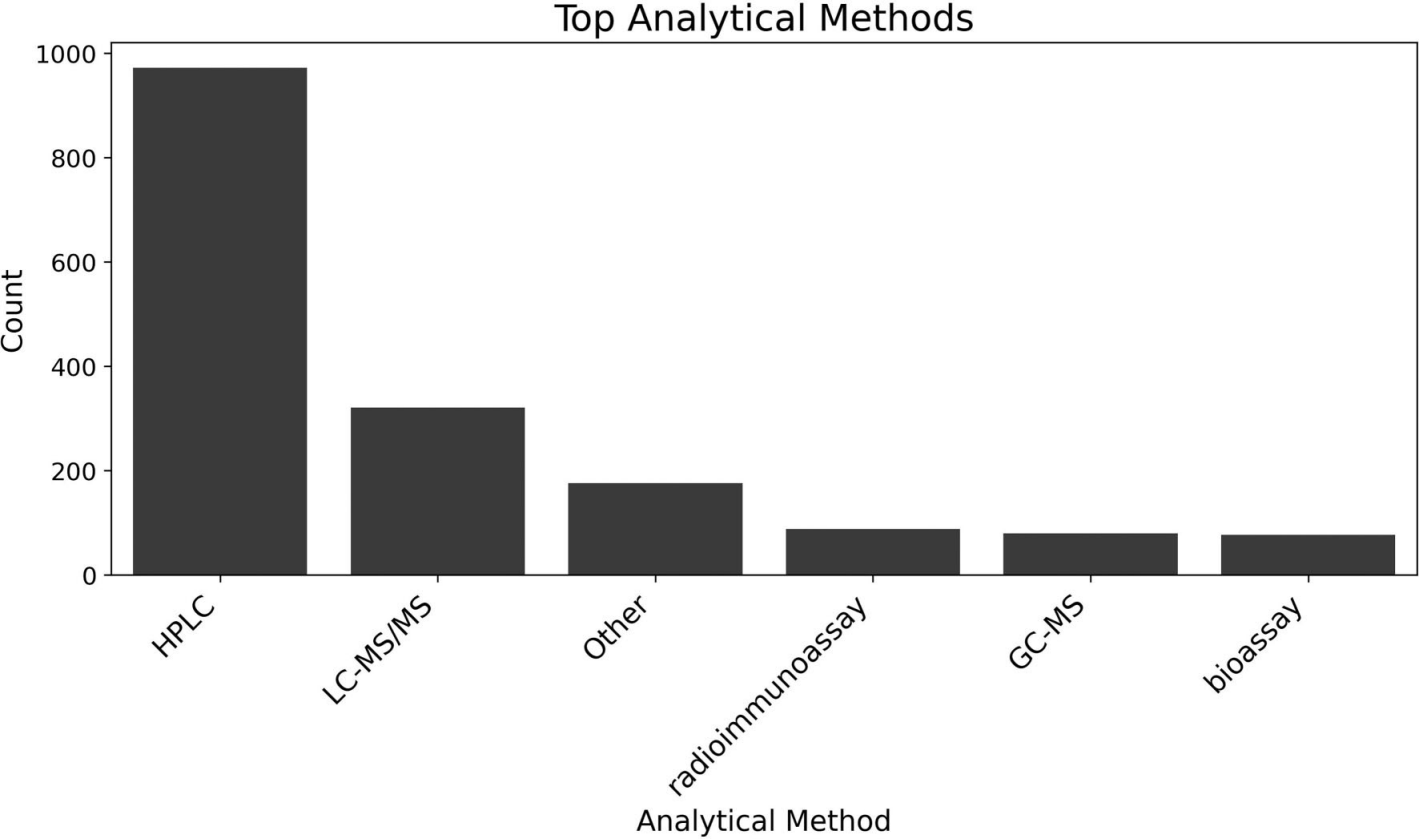
Counts of analytical methods presented in scientific articles. HPLC—high-performance liquid chromatography; LC-MS/MS—liquid chromatography–tandem mass spectrometry.

Regarding analytical techniques, the most frequently used methods were high-performance liquid chromatography (HPLC) and liquid chromatography–tandem mass spectrometry (LC-MS/MS). Figure 4 presents the distribution of the five most prevalent analytical methods, along with the ‘other’ category that includes other analytical methods.

The database is freely available in the Mendeley Data system (https://data.mendeley.com/datasets/3427×3wzzc/2).

## Discussion

The database presented here is the first publicly available, comprehensive source of information obtained entirely manually, without the use of automatic data collection tools. As a result, the collected data is characterized by high accuracy, consistency, and quality control, which distinguishes it from other existing pharmacokinetic resources. For comparison, the ChEMBL database [18] contains information on renal clearance in humans for 308 observations. However, these data were taken from a single publication by Varma et al., and they lack important experimental metadata such as study population size, dose administered, and route of administration [19]. Such limitations restrict the direct application of the dataset to advanced pharmacokinetic analyses and model development. Our database includes 154 compounds described in the Varma et al. study and additionally preserves detailed contextual information for each observed case.

The database can be used in both scientific research and clinical practice. The information it contains provides a solid basis for analyzing safe drug dosing, the effect of dose on glomerular filtration, and potential toxic effects in patients with renal impairment. Thanks to its precise development, this data can support the development of pharmacokinetic models, risk assessment, and the individualization of therapy in particularly sensitive populations.

Unlike the existing PK-DB database [20, 21], which only contains general information on clearance and does not specify renal clearance, the presented database contains data with a clear distinction regarding renal elimination. This is a significant advantage, enabling more accurate assessment of elimination processes dependent on kidney function and more reliable analysis of the safety of pharmacotherapy in this group of patients.

We acknowledge that within-compound data variability, for some drugs, is substantial. There are various potential reasons for this situation, beginning from expected between study variability, through application of various analytical methods, physiological and pathophysiological parameters etc. During this work, no attempt was made to speculate on the specific reasons for variability.

## Conclusions

In this study, we have developed a curated database containing experimentally determined values of human renal clearance, derived from scientific articles. The database contains over 1700 observations on more than 500 exogenous compounds, providing a structured and reliable source of information for pharmacokinetic studies. By integrating scattered data from multiple studies, this work fills an important gap in data availability and consistency. Although the database reflects the inherent heterogeneity of the experimental conditions described in different studies, it provides a valuable basis for future research.

## Supplementary Material

baag018_Supplemental_File
